# A Triton X-100-Based Microemulsion for the Removal of Hydrophobic Materials from Works of Art: SAXS Characterization and Application

**DOI:** 10.3390/ma11071144

**Published:** 2018-07-05

**Authors:** Michele Baglioni, Giovanna Poggi, Giulia Ciolli, Emiliano Fratini, Rodorico Giorgi, Piero Baglioni

**Affiliations:** Department of Chemistry and CSGI, University of Florence, via della Lastruccia, 3, 50019 Sesto Fiorentino (FI), Italy; baglioni_michele@csgi.unifi.it (M.B.); poggi@csgi.unifi.it (G.P.); ciolli@csgi.unifi.it (G.C.); fratini@csgi.unifi.it (E.F.); giorgi@csgi.unifi.it (R.G.)

**Keywords:** nanostructured fluid, cleaning, beeswax, paraffin, ceresin, oblate ellipsoid, wall paintings

## Abstract

The removal of hydrophobic materials from a porous support, such as wax stains on wall paintings, is particularly challenging. In this context, traditional methods display several drawbacks. The limitations of these methods can be overcome by amphiphile-based aqueous nanostructured fluids, such as micellar solutions and microemulsions. In this study, a microemulsion for the removal of wax spots from artistic surfaces was formulated. The nanostructured fluid includes a non-ionic surfactant, i.e., Triton X-100, and two apolar solvents, namely p-xylene and n-nonane. The solvents were selected on the basis of solubility tests of three waxes in several organic solvents. The nanostructured fluid was characterized by means of small-angle X-rays scattering (SAXS) and the information about micelle structure was used to understand the interaction between the microemulsion and the selected waxes. The microemulsion was then tested during the restoration of the frescoes in the Major Chapel of the Santa Croce Basilica in Florence, Italy. After some preliminary tests on fresco mockups reproduced in the laboratory, the nanostructured fluid was successfully used to clean some wax deposits from the real paintings, hardly removable with traditional physico-mechanical methods.

## 1. Introduction

The removal of hydrophobic materials from a porous support, such as unwanted and detrimental polymeric coatings and/or wax stains on stones or wall paintings, is a common procedure in the conservation of cultural heritage [1,2]. The removal of wax is particularly challenging due to the nature of this material. Conservators traditionally use apolar organic solvents [3,4], which swell or solubilize the wax, or physical methods, which usually soften the material by heating and subsequently allow the mechanical removal of the unwanted deposits. The major disadvantage of these methods is that the solved or molten wax is usually spread in the pores of the substrate, making any attempt of further removal even more complicated, if not impossible. Even if this can be tolerated, or even appreciated, from an aesthetic standpoint—since the wax optical density is reduced to such an extent that the stain could become invisible to the naked eye—, this effect should be firmly avoided. Some recent works have proposed the use of laser as a physical methodology to remove wax in a safer way [5,6]. The main drawback of laser cleaning is the requirement of expensive and not-easily portable instrumentation.

Amphiphile-based aqueous nanostructured fluids, such as micellar solutions and microemulsions, are nowadays valid alternatives to traditional cleaning methodologies in these contexts [1,2,7,8,9,10]. Their use in the conservation of cultural heritage was proposed for the first time in the early 1990s for the removal of wax stains from the frescoes, by Masaccio and Masolino, in the Brancacci Chapel in Florence, Italy [1,7]. These systems, which are mainly water-based, swell and detach polymeric coatings from hard surfaces, avoiding the spreading and redeposition of the removed matter. The mechanisms involved in these processes are currently under investigation [11,12,13,14,15,16]. If the unwanted material is composed of small molecules, solubilization or emulsification processes can take place [17,18,19], even if they are not commonly observed during the application of nanostructured fluids in the cleaning of artworks. Furthermore, the application of these systems is usually significantly safer and more controlled than unconfined organic solvents, in terms of work of art safeness and operators’ health [1,2]. Nanostructured fluids have reduced toxicity and enhanced eco-compatibility, which should make them even more appreciated by restorers and conservators.

In this paper, we prepared a new microemulsion for the removal of wax spots, which features Triton X-100 as surfactant and p-xylene and n-nonane as solvents. The solvents were chosen on the basis of solubility tests performed on three kinds of wax, previously characterized by FT-IR investigation. Triton X-100 was selected, as it is one of the most studied nonionic surfactants [20,21,22,23,24]. The use of a nonionic surfactant represents an improvement over previous formulations for the same purpose, as the obtained system is insensitive to the presence of ions, such as Ca^2+^ or Mg^2+^, which are frequently encountered in the cleaning of wall paintings and stones, and which could precipitate ionic surfactants, such as SDS in the form of insoluble salts. The characterization of the Triton X-100-based system was investigated by means of small-angle X-rays scattering (SAXS) before and after the addition of solvents. Information about micelle structure can be key to understanding the interaction between the microemulsion and the selected waxes.

Finally, the microemulsion was used in the context of a real and highly relevant conservation case study: the cleaning of the frescoes in the Major Chapel of the Santa Croce Basilica in Florence, Italy. These wall paintings were stained by several deposits of an unknown waxy material, which was sampled and characterized. After some preliminary tests on fresco mockups reproduced in the laboratory, the microemulsion was successfully used to clean some wax deposits from the real paintings, hardly removable with traditional physico-mechanical methods.

## 2. Materials and Methods

### 2.1. Chemicals

Triton X-100 (TX100, Sigma-Aldrich, Saint Louis, MO, USA, Purity > 95%), p-xylene (Merck, Darmstadt, Germany, purity > 99.5%), n-nonane (Sigma-Aldrich, purity > 99%), ligroin (Sigma-Aldrich, purity > 99%), d-limonene (Sigma-Aldrich, purity > 99.5%), turpentine (Fidea s.p.a., Matelica (MC), Italy, purity N.A.), chloroform (Sigma-Aldrich, purity > 99%) were used as received, without further purification. Beeswax, paraffin and ceresin were purchased from CTS, Italy, and used as received. Sand (50–70 mesh particle size) was purchased from Sigma-Aldrich, aged slaked lime was purchased from La Banca della Calce s.r.l., Bologna, Italy. Japanese paper (9.6 g/m^2^), artificial ultramarine blue pigment and cellulose powder (Arbocel^®^ BC200, J. Rettenmaier & Sohne, Gmbh, Rosenberg, Germany) were purchased from Zecchi, Florence, Italy. Water was purified with a Millipore Milli-Q gradient system (resistivity > 18 MΩ cm).

### 2.2. Microemulsion Preparation

**Figure 1:**
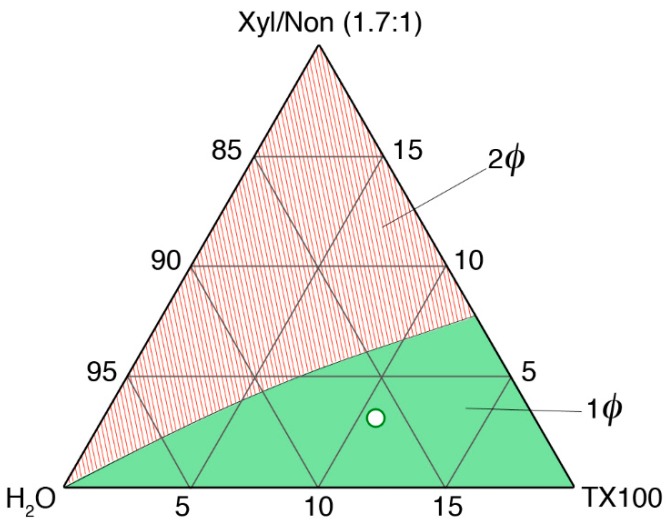
Investigated portion of the pseudo-ternary phase diagram of H_2_O/TX100/xylene:nonane (1.7:1 *w*/*w*) at 25 °C. The two-phase (red) and the single-phase (green) regions are evidenced. The white spot indicates the position of the microemulsion here reported in the phase diagram.

The microemulsion was prepared by adding under constant stirring the following components (% *w*/*w*): water, 86.2%; TX100, 10.7%; p-xylene, 1.8%; nonane, 1.3%. The system was equilibrated until an optically clear liquid was obtained. The amount of solvents was chosen by exploring the pseudo-ternary (water, TX100, xylene/nonane 1.7:1 molar ratio) phase diagram of the system (reported in Figure 1), in order to maximize solvents content while being sufficiently far from any phase separation boundary at room temperature.

### 2.3. Solubility Tests

5 mg of each of the three selected waxes (beeswax, paraffin and ceresin) were immersed in 4 mL of the chosen organic solvents and left equilibrating without shaking or stirring. The vials were then observed over time and pictures were taken at the equilibrium. Then, 5 mg of the three waxes were immersed in 20 mL of the proposed microemulsion and the same procedure was followed.

### 2.4. ATR FT-IR Spectroscopy

ATR FT-IR (Attenuated Total Reflectance-Fourier Transform Infrared) analyses were performed using a Nexus 870 spectrometer (Thermo-Nicolet, Madison WI, USA). The instrument was interfaced with OMNIC software (Thermo-Nicolet) and equipped with an ATR device for attenuated reflectance measurements. A Mercury Cadmium Telluride—MCT detector was used to collect the signal in the 4000–650 cm^−1^ range. The spectra were collected as single-beam files as the sum of 128 scans with a resolution of 4 cm^−1^. Then, they were divided by the background signal and expressed as reflectance percentage (%*R*).

### 2.5. Small-Angle X-ray Scattering

Small-angle X-ray scattering (SAXS) measurements were performed with a HECUS S3-MICRO SWAXS-camera (Hecus XRS, Graz, Austria), equipped with a Hecus System 3 2D-point collimator (min divergence 0.4 × 0.9 mrad^2^) and two PSD-50M position sensitive detectors (M. BRAUN, Garching, Germany) consisting of 1024 channels with a width of 54 μm. During the experiments, the *K_α_* radiation (*λ* = 1.542 Å) emitted by a Cu anode from the Oxford 50 W microfocus source with customized FOX-3D single-bounce multilayer point focusing optics (Xenocs, Grenoble, France) was used, while the *K_β_* line was removed by a multilayer filter. The voltage is generated by the GeniX system (Xenocs). The sample-to-detector distance was 26.9 cm. The volume between the sample and the detector was kept under vacuum during the measurements to minimize the scattering from the atmosphere. The camera was calibrated in the small-angle region using silver behenate (*d* = 58.38 Å). Scattering curves were obtained in the *q*-range between 0.003 and 0.6 Å^−1^. The temperature control was set to 25 °C. Samples were contained in 1.5 mm thick quartz capillary tubes sealed with hot-melting glue. Scattering curves were corrected for the empty capillary contribution considering the relative transmission factors. Desmearing of the SAXS curves was not necessary, thanks to the sophisticated focusing system.

### 2.6. Laboratory Cleaning Test

For laboratory cleaning tests, mortar sample tiles were prepared by mixing one part of slaked lime and three parts of sand. The mixture was then poured into wooden molds of 5 cm × 5 cm × 1.5 cm and then painted with an artificial ultramarine blue pigment using the fresco technique. The specimens were left untouched for a month, in order to reach the complete setting. Each sample was then stained by brushing some molten beeswax (diluted with ligroin) on the surface, in order to coat three quarters of the whole tile surface. Laboratory wax removal tests were then executed by using cellulose pulp powder as the vehicle for the cleaning fluid. Cellulose pulp poultices soaked with the microemulsion were applied over a Japanese paper sheet, which was interposed between the specimen surface and the cleaning system. The poultices were then covered with a polyethylene film in order to avoid fast evaporation of the microemulsion and applied for 2 h. The results of the cleaning tests were evaluated by photographic observation under UV light, comparing pictures taken before and after the treatment with the microemulsion.

### 2.7. In Situ Cleaning Test

Wax removal tests performed in the Major Chapel of the Santa Croce basilica, Florence, Italy, in the framework of the restoration of the fresco paintings, were carried out using the same procedure as for the laboratory tests. A cellulose pulp poultice soaked with the microemulsion was applied on the surface of the painting, interposing a Japanese paper sheet in between. The compress was applied for 2.5 h and then a gentle mechanical action was performed with a wet cotton swab, in order to remove the swollen wax.

## 3. Results and Discussions

### 3.1. Characterization of Waxes

Waxes are organic lipophilic moldable substances having a quite low melting point (between 40 and 75 °C). Animal waxes, such as beeswax, usually contain long-chain alkanes and lipids, together with fatty acids and long-chain alcohols. On the other hand, paraffin wax, which is a petroleum-derived wax, is a mixture of hydrocarbon molecules containing between twenty and forty carbon atoms. Refined mineral waxes, such as ceresin, obtained from ozokerite by a purifying process, usually consists of long alkyl chains [25].

**Figure 2:**
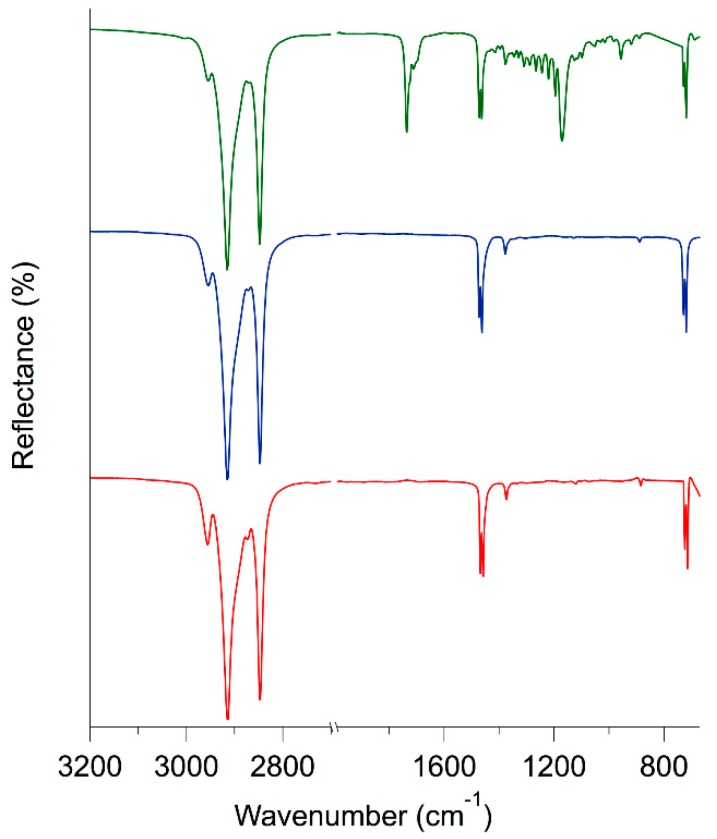
FTIR-ATR spectra of three selected waxes: beeswax (green), paraffin (blue) and ceresin (red).

FTIR-ATR spectra of beeswax, paraffin and ceresin are reported in Figure 2. As expected, the spectra of ceresin and paraffin are very similar, being their composition the same. In particular, signals due to the asymmetric and symmetric stretching of aliphatic hydrocarbons can be seen at 2915 and 2848 cm^−1^, respectively. The in-plane vibrations of aliphatic hydrocarbons are located at 1471, 1464 and 1375 cm^−1^, while the peak due to the rocking of the same vibrational group is found at 720 cm^−1^ [26,27]. As expected, in the FTIR-ATR spectrum of beeswax, all the signals due to aliphatic chains are present. In addition to that, two intense absorption bands can be seen at 1736 cm^−1^ (with a shoulder at 1710 cm^−1^) and 1172 cm^−1^. These are clearly related to the presence of carboxyl groups of fatty acids and esters [26,27]. In fact, beeswax usually contains about 14% of hydrocarbons, 67% of different types of esters and 12% of fatty acids [28].

The solubility of the three waxes was tested by immersing 5 mg of each product in vials containing 4 mL of the following organic solvents: ligroin, n-nonane, p-xylene, limonene, turpentine and chloroform. These solvents were chosen according to their theoretical solubility parameters. In fact, they should be good solvents for highly hydrophobic materials, such as wax. Chloroform was selected as reference, as it is one of the most efficient solvents, but in the context of conservation of cultural heritage it should be avoided, when possible, due to its toxicity.

As can be seen from the solubility results reported in Table 1, the three waxes behave differently and the selected solvents also have different effectiveness in solubilizing the different products.

Beeswax and ceresin are hardly soluble, while paraffin is easily solubilized by all the tested solvents. In particular, ceresin swells in all the solvents, while the behavior of beeswax is more varied; it is emulsified by ligroin and nonane and swollen by limonene and turpentine. An interesting consideration can be drawn by comparing paraffin and ceresin, which, from a compositional standpoint, had almost identical results, according to the FT-IR investigation. Nevertheless, their behavior in solvents is completely different, with paraffin being much more soluble than ceresin. Despite having the same chemical composition, ceresin mostly consists of branched-chain and closed-chain hydrocarbons. In paraffin, the hydrocarbon chains are mostly linear [25]. This could explain the different behavior of the two waxes when immersed in the selected organic solvents.

According to solubility tests results, nonane and xylene were selected for the removal of these waxes. Ligroin could have been chosen, as it proved as good as nonane, but it was avoided, since, being a mixture of solvents, it would have complicated the structural characterization of the microemulsion. On the other hand, as anticipated, chloroform is a good solvent for wax, but its use was excluded due to health concerns.

Nonane and xylene were then used to formulate an o/w microemulsion based on Triton X-100 as the surfactant.

### 3.2. Formulation and Characterization of the Microemulsion

The microemulsion was formulated and prepared according to what was reported in Section 2.2. A SAXS characterization of this system was performed in order to investigate the nanostructure of micellar aggregates both in the absence and in the presence of solvents. Therefore, the following samples were analyzed: H_2_O/TX100, H_2_O/TX100/xylene, H_2_O/TX100/xylene/nonane, i.e., the complete microemulsion. The *w*/*w* ratio between the components was kept constant from one sample to another. TX100 micelles in water have been quite extensively studied in the past, and in the literature, several models can be found for the structural description of such supramolecular aggregates, going from spherical to variously ellipsoidal particles [20,21,22,23,24,29,30,31]. However, the most widely accepted hypothesis is that TX100 micelles in water are not spherical; rather, they have the shape of oblate ellipsoids [20,21,22,23,24,29,32].

According to these premises, we fitted our data using a NIST fitting model [33], which calculates the scattering intensity as given by the form factor for an oblate ellipsoid particle with a core/shell structure. The form factor is averaged over all possible orientations of the ellipsoid, such that

(1) $$P\left(q\right)=\mathrm{scale}\times \frac{<f^{2}>}{\mathrm{Vol}}+\mathrm{bkg}$$

where <*f*> is the orientationally averaged single particle scattering amplitude. The function calculated is:

(2) $$P\left(q\right)=\frac{\mathrm{scale}}{V_{shell}}\int_{0}^{1}{\left|F\left(q,a,b,\alpha \right)\right|}^{2}d\alpha +\mathrm{bkg}$$

(3) $$F\left(q,a,b,\alpha \right)=3\left(\rho_{core}-\rho_{shell}\right)\frac{V_{core}j_{1}\left(u_{core}\right)}{u_{core}}+3\left(\rho_{shell}-\rho_{solv}\right)\frac{V_{shell}j_{1}\left(u_{shell}\right)}{u_{shell}}$$

where:

(4) $$V_{core}=\left(\frac{4\pi }{3}\right)ba^{2}$$

(5) $$V_{shell}=\left(\frac{4\pi }{3}\right)\left(b+t\right){\left(a+t\right)}^{2}$$

(6) $$u_{core}=q\sqrt{a^{2}\left(1-\alpha^{2}\right)+b^{2}\alpha^{2}}$$

(7) $$u_{shell}=q\sqrt{{\left(a+t\right)}^{2}\left(1-\alpha^{2}\right)+{\left(b+t\right)}^{2}\alpha^{2}}$$

And

(8) $$j_{1}\left(x\right)=\frac{\left(\sin x-x\cos x\right)}{x^{2}}$$

With *a* being the major semiaxis of the oblate ellipsoid, *b* the minor semiaxis of the ellipsoid (and the rotational axis), *t* the shell thickness, α an orientation variable, i.e., the cosine of the angle between the scattering vector and *a*, and *ρ*_core_, *ρ*_shell_ and *ρ*_solv_ the scattering length densities of the micelle core, micelle shell and of the solvent, respectively.

The returned value is the scattered intensity per unit volume, I(q) = f × P(q). No interparticle interaction is included in this calculation.

**Figure 3:**
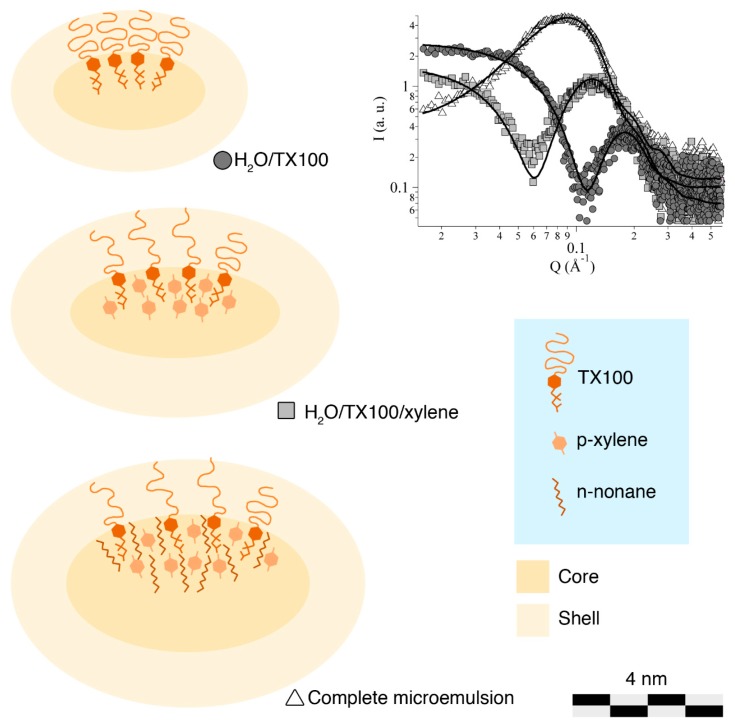
SAXS scattering profiles measured on three systems: H_2_O/TX100 (dark grey circles), H_2_O/TX100/xylene (light grey squares), complete microemulsion (white triangles). Fitting curves are reported as continuous black solid lines. The experimental errors are not reported here because they are smaller than the markers. The cartoon shows the evolution of the micelles (here reported as a vertical section) and the location of the components in the aggregates’ structure, as inferred by SAXS fitting results. Hydration water is not showed for sake of clarity. (Micelles reported here are about 8.7 million times larger than their actual size).

Figure 3 reports the scattering data, together with the fitting lines, and a cartoon illustrating the structural evolution of micellar aggregate, according to the fitting results reported in Table 2. The geometrical parameters for TX100 micelles in water are in good agreement both with the size of the surfactant molecule and with the data available in the published literature. In more detail, we obtained the same value as Robson and Dennis (1997) [23] for the rotational axis *b*, 10.4 Å, while, according to our fitting, the semiaxis *a* was about 20 Å, i.e., significantly shorter than the one obtained by them (34 Å). This could be due to the fact that their results were obtained as theoretical calculations based on molecular weight and intrinsic viscosity of TX100; therefore, some slight deviation from experimental data could be anticipated. On the other hand, in our fitting, the shell thickness, *t*, is 11 Å, which is shorter than the one hypothesized by Robson and Dennis (17 Å), but is in good accordance with the value reported by Goyal et al. (1995) [29], i.e., 12.5 Å, which is obtained from SANS experiments. We thus confirmed that TX100 micelles in water seem to have an oblate ellipsoidal shape. Again on the basis of the work of Robson and Dennis [23], a hydration number of ca. 34, which is to say that each polyethoxylate chain of TX100 interacts with an average of 34 water molecules, was hypothesized. The initial scattering length density of the shell (SLD_shell_) was calculated accordingly to that value of hydration. Upon the addition of xylene and, then, of nonane, TX100 micelles swell, as could be expected, while maintaining an oblate ellipsoidal shape. The core volume changes from 17.8 Å^3^ to 39.5 Å^3^ to 80.5 Å^3^, indicating that both xylene and nonane are located in the hydrophobic region of the aggregates. In addition to that, the shell thickness slightly increases when xylene is added to the system, while remaining almost constant for the complete microemulsion. Hydration water also increases, because of the minor density of polyethoxylate chains in the swollen shell. Accordingly, the value of SLD_shell_ decreases upon solvent addition.

**Figure 4:**
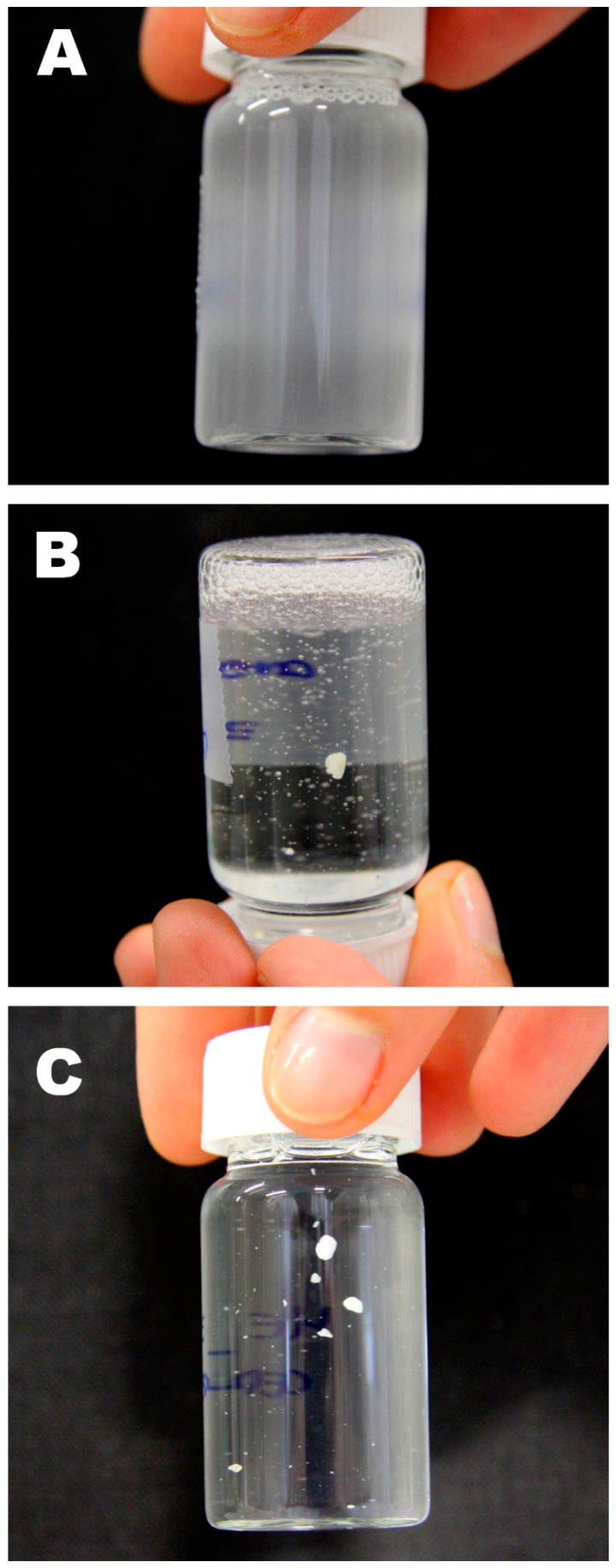
(**A**) 5 mg of paraffin emulsified in 20 mL of microemulsion; (**B**) 5 mg of beeswax swollen by 20 mL of microemulsion; (**C**) 5 mg of ceresin swollen by 20 mL of microemulsion.

Figure 4 shows the solubility tests of wax, i.e., paraffin and beeswax, in the formulated microemulsion. Paraffin is emulsified (Figure 4A), while beeswax is only swollen (Figure 4B) by the nanostructured fluid. This can be explained by the chemical composition of the two waxes and the structure of TX100/xylene/nonane micelles. The linear alkyl molecules of paraffin behave similarly to nonane and can be completely or partially solubilized in the hydrophobic core of TX100 swollen micelles (see Figure 3), resulting in the complete emulsification of the material in the nanostructured fluid. This system is no longer a microemulsion, and its cloudy appearance indicates that swollen micelles and/or nanodroplets of swollen wax are larger than a few hundreds of nanometers. On the other hand, beeswax being mainly composed of esters, its solubilization or emulsification is much harder. In fact, while linear esters in principle could fit inside the hydrophobic core, di- and triglycerides of fatty acids are molecules that are too large to be solubilized into TX100 micelles. Therefore, beeswax is only swollen by xylene and nonane, which migrate from micelles to the bulk wax. Similarly, as reported in Figure 4, ceresin is swollen by the tested nanostructured fluid. It is worth recalling that, despite having the same chemical composition, ceresin mostly consists of branched-chain and closed-chain hydrocarbons, while, in paraffin, the hydrocarbon chains are mostly linear [25]. SAXS measurements on the microemulsion recovered after the interaction with beeswax were performed in order to investigate the structure of micelles depleted in organic solvents. As expected, the size of the micelles was slightly reduced, accounting for the partial migration of the organic solvents from the microemulsion to the wax. Scattering data, however, were almost superimposable to the ones of the pristine microemulsion, and thus are not shown here for the sake of clarity and conciseness. On the other hand, unfortunately, it was not possible to perform SAXS measurements on emulsified paraffin, as the obtained system was unstable.

These tests show that the proposed microemulsion is able to effectively interact with wax, inducing the complete emulsification or the swelling of the material. This result was a promising premise to further tests performed on wall paintings mock-ups and real works of art.

### 3.3. Wax Removal Tests

As for most Christian churches, in the Major Chapel of the S. Croce basilica in Florence, votive wax candles were kept for centuries close to the frescoes that decorate the walls. Given that these candles were mostly blown out by people, several wax spots and stains are nowadays present on the wall paintings in the form of aged and brownish concretions. In the frame of the restoration workshop of the frescoes of the Major Chapel, we tested our microemulsion for the removal of these wax deposits. Expert conservators from the Opificio delle Pietre Dure, Florence, had tried different physico-mechanical methods to remove the wax spots, but these were only partly successful.

**Figure 5:**
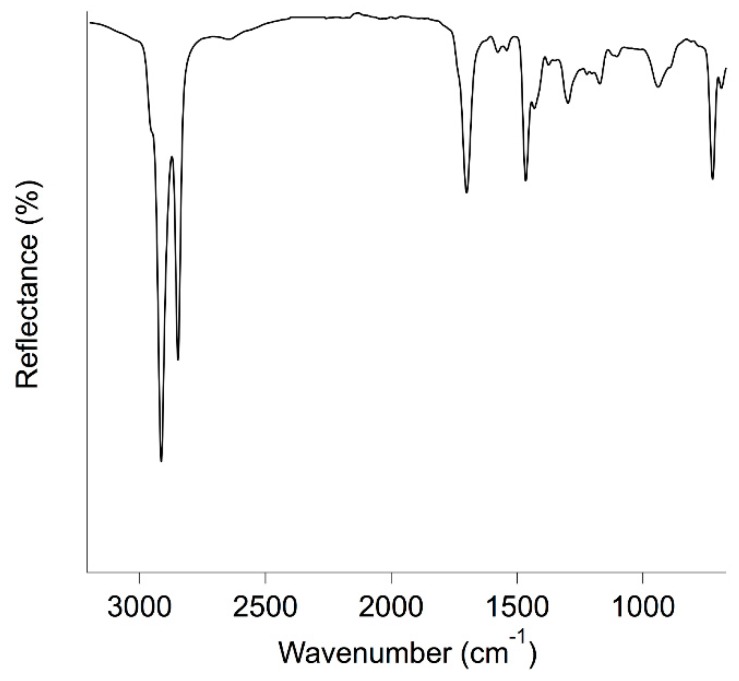
ATR spectrum of wax from Major Chapel of the S. Croce basilica, Florence.

In Figure 5, the FTIR-ATR spectrum of a wax sample removed from the fresco paintings of the Major Chapel of the S. Croce basilica is reported. The spectrum is similar to that of pristine beeswax, reported in Figure 2. In fact, the signals due to aliphatic chains can be seen at 2912 cm^−1^, 2846 cm^−1^, 1464 cm^−1^ and 723 cm^−1^, while several other peaks, due to carbon-oxygen bonds, are present in the fingerprint zone. The main difference between the two spectra relies in the position of the signal related to the vibration of C=O group. In fact, in S. Croce’s wax, this peak is located at 1700 cm^−1^ and has a shoulder at 1735 cm^−1^. This difference is due to the partial hydrolysis of esters, which transform into carboxylic acids during heating, increasing the total amount of free fatty acids in aged beeswax [34].

**Figure 6:**
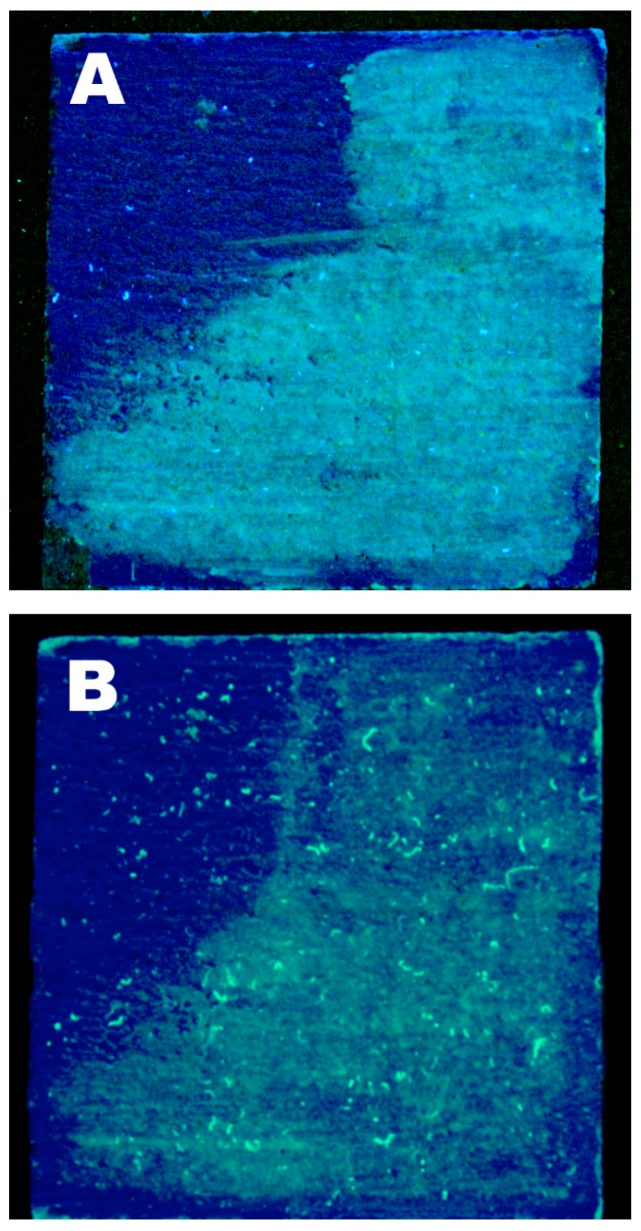
Wax removal tests performed in the laboratory. The picture shows one of the wax-stained tiles treated with the microemulsion (**A**) before and (**B**) after the cleaning. After the microemulsion application, the wax coating was thinned and partly removed. Some swollen wax was also moved on the surface, both by the microemulsion and by the subsequent mechanical action performed with a wet cotton swab. The luminescent spots in (**B**) are residues of cellulose fibers, due to the application of the cellulose powder poultices and could be almost completely removed by gently washing the surface with pure water after the cleaning.

Therefore, in order to try and reproduce the conservational scenario of the S. Croce basilica, fresco mockups were prepared in the laboratory and then stained with beeswax. It was not possible to exactly replicate the waxy concretions present on the real wall paintings, where the wax was likely altered and mixed with dirt, dust and unknown matter. At first, we tried to drip some molten beeswax on the surface of fresco tiles, but the obtained wax spots were easily removable by means of some gentle mechanical action, differently to what observed by conservators in the real case. Therefore, in order to test the microemulsion in the worst possible scenario, beeswax was heated, diluted with ligroin and brushed onto the tiles, so as to obtain a waxy coating (clearly visible in Figure 6, under UV light). It is worth saying that this situation is only indicative of the real conservational challenge faced by restorers in the Florentine church; however, cleaning tests performed on such samples provide useful information on the effectiveness of the microemulsion.

Figure 6 shows one of the cleaning tests, which is representative of the average results obtained. The wax coating is visible under UV light both before and after the application of the microemulsion-loaded compress (see Section 2.6 for the details on the used methodology). We knew from the solubility tests that we could not expect the microemulsion to solubilize or emulsify the beeswax. Thus, in this case, the best results would have been the swelling of the waxy coating, which could have been subsequently removed with mechanical action. In this sense, microemulsions grant a better control over the cleaning action than neat and unconfined organic solvents, which, as already stated, may solubilize the wax and further spread it into the porosity of the substrate. From the comparison between Figure 6A,B, it can be noticed that the wax coating is thinned and partly removed; however, the result is only partly satisfactory. In fact, most likely, a major part of the beeswax deposited on the fresco mockup penetrated in the pores of the mortar, since it was brushed on the surface while being molten and diluted in ligroin. It is worth noting that this is completely different from the real staining of frescoes due to the extinction of votive candles. In this case, the complete removal of wax is extremely difficult, besides not being so representative of most of the real conservation cases. This is one major issue that affected our cleaning tests results. In principle, the microemulsion application could be repeated several times, provided that the painting is capable of enduring more treatments on the same area, in order to try and further remove the remaining wax. However, in our case it was not relevant to repeat the application, mainly because of the limited representativeness of our samples with respect to the real conservative case.

**Figure 7:**
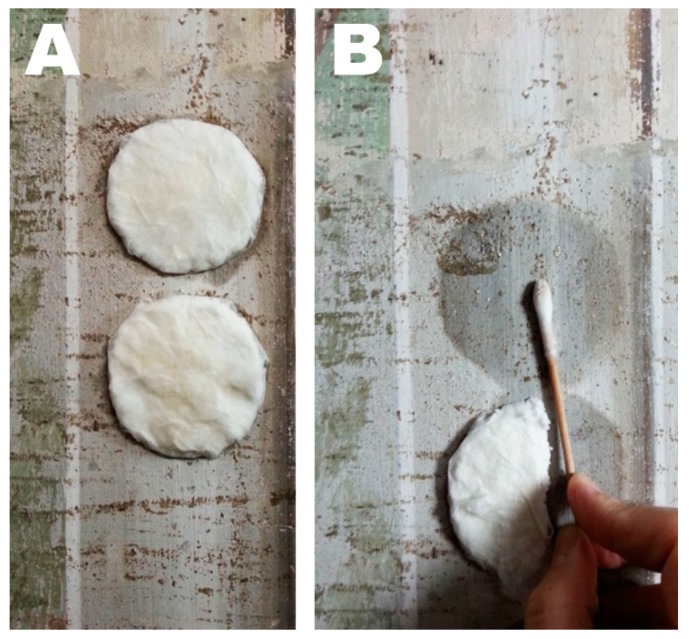
Wax removal tests performed in the Santa Croce Basilica, Florence, Italy. The wax stains appear as brownish concretions, which randomly cover the fresco surface. (**A**) Microemulsion-loaded compresses during the 2.5 h application. (**B**) Mechanical removal of the swollen and softened wax deposits by means of a humid cotton swab. Wax removal is evidenced by the brownish staining of the cotton swab tip.

Thus, acknowledging the positive result—though not completely satisfactory—of laboratory cleaning tests, the microemulsion was tried in the removal of wax concretions from the frescoes of the S. Croce basilica (Figure 7). In this case, the nanostructure fluid gave excellent results, better than the traditional physical methods previously tested by conservators. The waxy stains were swollen and softened, and they were easily removed by means of a gentle mechanical action performed with a wet cotton swab (see Figure 6B, the swab shows traces of the brownish matter removed). Unfortunately, it was not possible to perform analytical measurements on the treated areas; thus, the presence of traces of residual wax cannot be excluded. However, we found that the proposed cleaning system was even more effective on the real case than in the laboratory tests.

## 4. Conclusions

In conclusion, we proposed a new o/w microemulsion based on Triton X-100, xylene and nonane for the removal of hydrophobic matter (i.e., mainly wax) from works of art. We studied the chemical composition and the solubility of three waxes and found that apparently similar materials show significant differences in their response to the action of solvents. On the basis of these tests, the solvents for the formulation of the microemulsion were chosen, which proved to be capable of emulsifying paraffin and swelling beeswax and ceresin. SAXS characterization of the system confirmed that, as reported in the literature, Triton X-100 micelles in water have the shape of oblate ellipsoids and maintain this shape also after the addition of xylene and nonane to the water/surfactant binary system, showing that these solvents just slightly swell the supramolecular aggregates without causing major changes in the nanofluid structure. The different behavior of waxes in solvents was clarified by the nanostructure of Triton X-100 micelles, which are too small to permit the solubilization of di- and triglycerides (beeswax) or branched hydrocarbon (ceresin) into their hydrophobic core. The linear alkyl chains of paraffin, on the other hand, are solubilized in the hydrophobic core of TX100 swollen micelles, resulting in the emulsification of the wax in the microemulsion. Finally, we tested the new microemulsion for the removal of wax concretions from the frescoes of the Major Chapel in the Santa Croce basilica, Florence, Italy. The undesired matter was sampled and identified as mainly composed of beeswax. Fresco mockups were, thus, prepared and stained with wax obtaining samples that were only partly representative of the real case. The water/Triton X-100/xylene/nonane microemulsion gave partly satisfactory results in laboratory cleaning tests, while it proved extremely effective on the real case, giving excellent cleaning results. In general, when a hydrophobic coating has to be removed from a porous hydrophilic substrate, such as a wall painting or a stone, the use of neat unconfined organic solvents poses some issues. The coating can be dissolved, and the solved matter tends to migrate inside the pores, where it can be re-deposited and its further removal becomes virtually impossible. Using microemulsions (or, more generally, water-based nanostructured systems), this issue is avoided, due to the different cleaning mechanism with respect to neat unconfined organic solvents. In particular, the hydrophobic coating is usually swollen, softened, detached or dewetted from the surface, without being dissolved, and this is enough to prevent it from migrating and re-depositing into the pores. In the present case, the beeswax (both in the lab and in situ) was visibly only swollen, so that there was no need to perform further analyses, in order to check for possible wax residues inside the porous matrix due to the cleaning action. We demonstrated, thus, that microemulsions can be versatile and effective tools for restorers, which can use them as an alternative to traditional methods, even in complex conservational challenges. Furthermore, knowing their nanostructure, i.e., performing a thorough physico-chemical characterization, is key to explaining and anticipating behavior, cleaning mechanisms and detergent properties of these novel cleaning systems.

## Figures and Tables

**Table 1:** Results of solubility tests performed on the three selected waxes.

Wax	Solvents					
	Ligroin	n-Nonane	p-Xylene	Limonene	Turpentine	Chloroform
Beeswax	Emulsified	Emulsified	Solubilized	Swollen	Swollen	Solubilized
Paraffin	Solubilized	Solubilized	Solubilized	Solubilized	Solubilized	Solubilized
Ceresin	Swollen	Swollen	Swollen	Swollen	Swollen	Swollen

“Solubilized” = the wax completely disappears, to the naked eye, and the solvent is optically clear as before; “Emulsified” = the wax disaggregates into very small pieces of swollen material and, rather than a real solution, a cloudy dispersion is obtained; “Swollen” = the wax piece swells, while keeping almost unaltered its appearance. The solving power of solvents towards a given wax was evaluated as follows: solubilization > emulsification > swelling.

**Table 2:** SAXS fitting parameters for the three systems analyzed.

	Systems		
Fitting Parameters	H_2_O/TX100	H_2_O/TX100/xylene	Microemulsion
*a* (Å)	20.2 ± 0.2	27.9 ± 1.3	32.4 ± 0.1
*b* (Å)	10.4 ± 0.2	12.1 ± 2.4	18.3 ± 0.1
*t* (Å)	11.1 ± 0.1	15.8 ± 3.3	14.7 ± 0.2
SLD_core_ (10^−6^ Å^−2^)	7.8	7.8	7.8
SLD_shell_ (10^−6^ Å^−2^)	10.8	10.0	9.9
SLD_solvent_ (10^−6^ Å^−2^)	9.4	9.4	9.4

*a* is the major semiaxis of the oblate ellipsoid; *b* is the minor semiaxis of the ellipsoid (and the rotational axis); *t* is the shell thickness. The scattering length densities (SLD) were calculated and kept constant during the fitting.
